# Axially connected nanowire core-shell p-n junctions: a composite structure for high-efficiency solar cells

**DOI:** 10.1186/s11671-015-0744-3

**Published:** 2015-01-28

**Authors:** Sijia Wang, Xin Yan, Xia Zhang, Junshuai Li, Xiaomin Ren

**Affiliations:** State Key Laboratory of Information Photonics and Optical Communications, Beijing University of Posts and Telecommunications, No. 10. Xitucheng Road, Beijing, 100876 China

**Keywords:** Solar energy, Nanophotonics and photonic crystals, Semiconductors, 88.40.H-, 88.40.jp, 81.07.Gf

## Abstract

A composite nanostructure for high-efficiency solar cells that axially connects nanowire core-shell p-n junctions is proposed. By axially connecting the p-n junctions in one nanowire, the solar spectrum is separated and absorbed in the top and bottom cells with respect to the wavelength. The unique structure of nanowire p-n junctions enables substantial light absorption along the nanowire and efficient radial carrier separation and collection. A coupled three-dimensional optoelectronic simulation is used to evaluate the performance of the structure. With an excellent current matching, a promising efficiency of 19.9% can be achieved at a low filling ratio of 0.283 (the density of the nanowire array), which is much higher than the tandem axial p-n junctions.

## Background

The development of high-efficiency photovoltaic (PV) systems has been a topic of intensive research recently. Most efficient solar-energy-harvesting devices are fabricated using compound III-V semiconductor materials in an advanced multi-junction structure [1-3]. However, the higher costs of III-V materials, increased complexity, and manufacturing price have been inhibiting the commercial application of multi-junction solar cells. A possible solution of this problem is to adopt III-V semiconductor nanowires (NWs) for solar cells.

NWs exhibit excellent properties for PV applications. It has been widely demonstrated that NWs with a well-defined geometrical structure can achieve higher light absorption than their thin-film counterparts with reduced material use, by combining intrinsic anti-reflection and enhanced light trapping [4-9]. In addition, the unique geometry of the NW enables the formation of advanced heterostructures, which can improve the performance of solar cells substantially. For example, benefiting from the highly effective lateral stress relaxation at the NW sidewall [10,11], high-quality p-n junctions with different bandgaps can be stacked into a single NW, which dramatically expands the absorption spectrum. Another promising structure is the core-shell p-n junction, which enables substantial light absorption along the long axis of the NW and efficient radial carrier separation and collection [12,13]. Furthermore, the pure ‘axial’ or ‘radial’ tandem solar cell also has its limit. Because the photogeneration events happen most frequently at the center of NWs, the axial junction cannot intrinsically block the generated carriers from reaching the surface and recombining like the radial junctions. Due to a similar reason, the radial tandem solar cell faces a challenge in efficient absorption for the shell junctions away from the core of NWs. Thus, it is not difficult to imagine that a composite structure which combines the advantages of the ‘axial’ and ‘radial’ structures would provide much higher efficiency compared with homogeneous ones.

In this paper, we propose a novel NW composite nanostructure for high-efficiency solar cells. The structure consists of several core-shell p-n junctions with different III-V materials which are axially connected by the tunnel diode in a NW. The stacked subcells with different bandgaps can exploit the solar spectrum very effectively. The core-shell p-n junction in each subcell provides an efficient collection of photogenerated carriers, which leads to a high photocurrent. A coupled three-dimensional (3-D) optoelectronic simulation is used to explore the photovoltaic efficiency of the structure. Finite-difference time-domain (FDTD) method is employed to investigate the light absorption characters and light trapping effects of the NW array. After the optimization, a high conversion efficiency of 19.9% is obtained at a low filling ratio of 0.283 (the filling ratio is defined as the area ratio between the total top surface of the NW arrays and the substrate), which is much higher than that of the tandem axial p-n junction counterpart.

## Methods

### Device design

**Figure 1:**
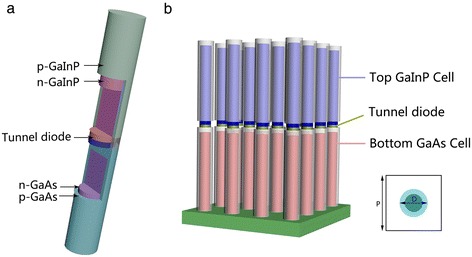
**3-D illustration and schematic drawing of the proposed structure. (a)** 3-D illustration of the proposed axially connected core-shell structure: core-shell p-n junctions with different III-V materials are axially connected by the tunnel diode in a NW. **(b)** Schematic drawing of vertically aligned NW arrays.

**Figure 2:**
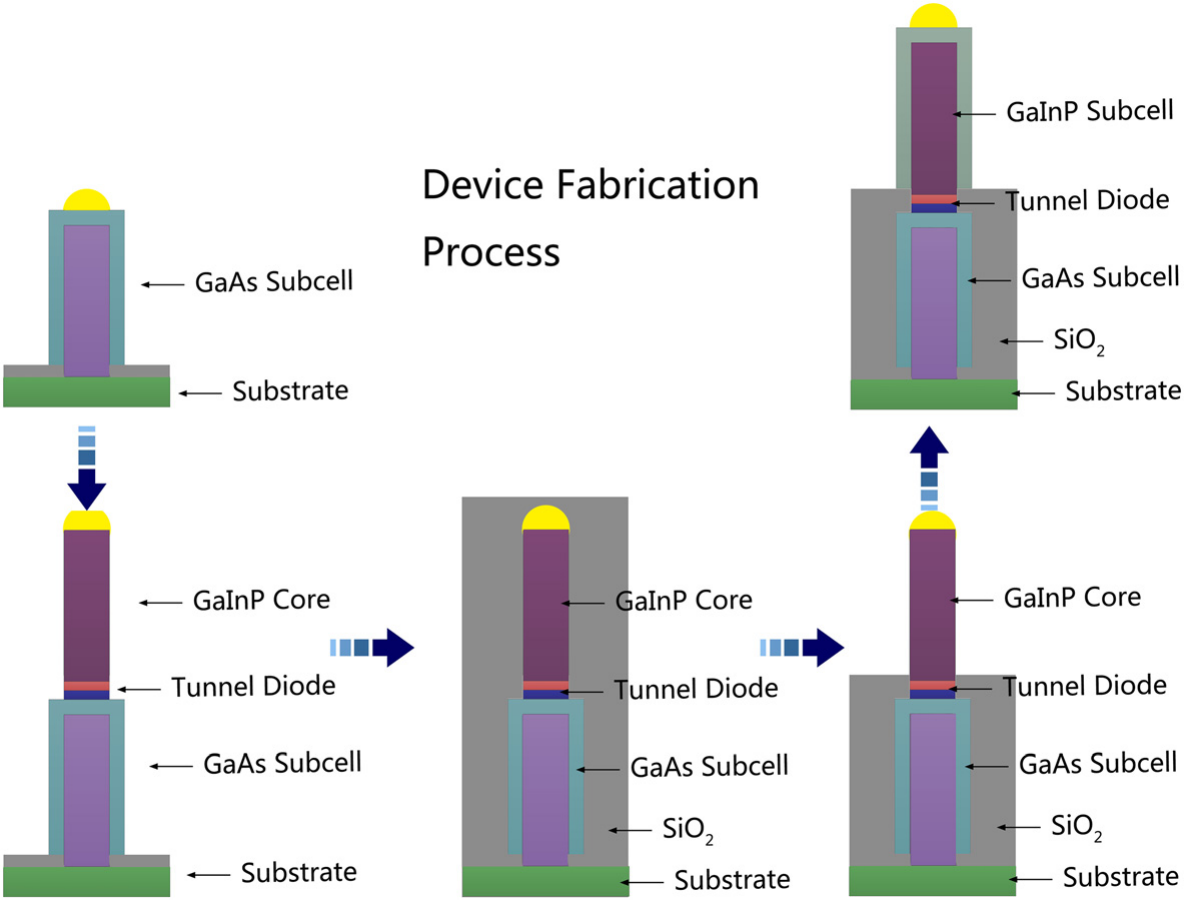
**The fabrication process of the proposed structure.** After growing the tunnel diode and GaInP core nanowire, we mask the device by SiO_2_, and then the SiO_2_ mask is etched to the position which is a little higher than where the tunnel diode lies, in order to isolate GaInP shell from the tunnel diode in electrics.

The schematic of the structure is shown in Figure 1a. Each subcell consists of an n-type NW core encapsulated in a p-type NW shell, which can be achieved by controlling the growth temperature to realize the switch between the core shell and axial growth [14-17]. The growth of GaAs nanowire arrays can be achieved using gold seed particles in arrays which can be arranged by a nanoimprint lithography. The core diameter and the period of arrays can be controlled by tuning the sizes and array pitches of the Au seeds [18]. The detailed fabrication process is shown in Figure 2. Different subcells can be connected by using a p^++^-n^++^ tunnel diode which bridges the p-shell of lower subcell and n-core of the subcell above. The high-performance single nanowire tunnel diodes have been reported recently [19]. Thus, the composite structure is practically achievable via traditional vapor-liquid-solid (VLS) growth method [20,21]. In the simulation, the top cell is composed of Ga_0.5_In_0.5_P with a bandgap of 1.8 eV, while GaAs with a bandgap of 1.42 eV is chosen for the bottom cell, for the purpose of absorbing the incident sunlight sufficiently with a practical lattice-matching. For each subcell, a core-shell p-n junction configuration is employed with a n-type core NW and a p-type shell NW, which are uniformly doped to 1 × 10^17^ and 3 × 10^18^ cm^−3^, respectively, while the doping concentration of the n-type GaAs substrate is 1 × 10^17^ cm^−3^. The length *L* of each subcell is fixed to 2 um, which is comparable to the film thickness in III-V solar cells. As shown in Figure 1b, the tandem NWs are arranged in a square array, where the *D*/*P* ratio determined by the period of the square lattice (*P*) and the diameter of NWs (*D*) is set to 0.5. For small *D*/*P* ratio, the incident light cannot be coupled into the NW and absorbed efficiently, resulting in a decrease in the conversion efficiency. However, higher *D*/*P* ratio means more consumption of Ga and In element, which are not found abundant in the earth crust and thus very expensive. Meanwhile, the efficiency does not always increase with increasing *D*/*P* ratio and has a upper limit. Thus, we select a relative low *D*/*P* ratio of 0.5 to achieve a good balance between the light absorption and material consumption [22]. The tunnel diode is assumed to be ideal (i.e., resistive or optical absorption losses are neglected) [23]. To isolate the GaInP shell from the tunnel diode in electrics, a transparent dielectric material, e.g., SiO_2_ is placed around the tunnel diode.

### Simulation method

Optical properties of the structure are investigated using Sentaurus Electromagnetic Wave (EMW) Solver module package. The axially connected NW core-shell p-n junctions are theoretically analyzed by using 3-D FDTD simulations [24-26]. The minimum cell size of the FDTD mesh is set to 5 nm, and the number of nodes per wavelength is 20 in all directions. By placing periodic boundary conditions, the simulations can be carried out in a unit cell to model the periodic array structure. A perfect matching layer (PML) is used under the GaAs substrate to assume semi-infinite substrates for simplified computation. The wavelength-dependent complex refractive index used in the simulations is obtained from the SOPRA N&K Database [27]. Normally, incident light is defined with power intensity and wavelength values from a discretized AM 1.5G solar spectrum. The AM 1.5G spectrum is divided into 62 discrete wavelength intervals, from 290 to 900 nm. The corresponding unpolarized feature of sunlight is modeled by superimposing the transverse electric (TE) and transverse magnetic (TM) mode contributions. To obtain the total optical generation under AM 1.5G illuminations, the power-weighted photogeneration rates of a single wavelength are superimposed from the FDTD simulation results [28,29]. The optical generation rate G_ph_ is obtained from the Poynting vector S:

1 $$ {G}_{\mathrm{ph}}=\frac{\left|\overrightarrow{\nabla}\cdot \overrightarrow{S}\right|}{2\hslash \omega }=\frac{\varepsilon^{{\prime\prime} }{\left|\overrightarrow{E}\right|}^2}{2\hslash } $$

where *E* is the electric field intensity at each grid point, *ω* is the angular frequency of the incident light, *ℏ* is the reduced Planck's constant, and *ε''* is the imaginary part of the permittivity.

For the electrical modeling, the 3-D optical generation profiles are incorporated into the finite-element mesh of the NWs in the electrical tool [29]. The device electrical simulation takes the doping-dependent mobility (GaAs only) and bandgap narrowing, radiative, Auger and Shockley-Reed-Hall (SRH) recombinations into consideration. The material parameters critical for device simulations are mostly obtained from the Levinshtein model [30], which is shown in Table 1. The Arora model [29,31] is adopted in the calculation of the doping-dependent mobility, which reads:

**Table 1:** **Key material parameters** ^**a**^

**Parameters**	**Values**	
Minimum mobility (cm^2^/V · s)	2.136 × 10^3^ (21.48)	2.3 × 10^3^ (1.12 × 10^2^)
SRH lifetime (ns)	1 (1)	10 (10)
Effective density of states (/cm^3^)	4.42 × 10^17^ (8.47 × 10^17^)	1.17 × 10^18^ (1.98 × 10^19^)
Auger coefficient (cm^6^/s)	1.9 × 10^−31^ (1.2 × 10^−31^)	3.0 × 10^−30^
Surface recombination velocity (cm/s)	10^7^ (10^7^)	10^7^ (10^7^)

^a^Unless mentioned specifically, all simulations in this work use the parameters in this table by default. The numbers in the front and in the parentheses are for the electrons and holes, respectively.

2 $$ {\mu}_{\mathrm{dop}}={\mu}_{\min }+\frac{\mu_d}{1+{\left(N/{N}_0\right)}^A} $$

where *A* is 0.6273 (0.8057) and *N*_0_ is 7.345 × 10^16^ (5.136 × 10^17^)/cm^3^ for the electrons (holes). The current-voltage relationship which is calculated by:

3 $$ J={J}_{\mathrm{sc}}-{J}_0\left({ \exp}^{V/{V}_c}-1\right) $$

where *J* is the current density of the solar cell, *J*_sc_ is the photocurrent density, *J*_0_ is the reverse saturation current density, and *V* is the voltage between the terminals of the cell. *V*_c_ is the thermal voltage, which can be given by:

4 $$ {V}_{\mathrm{c}}=\frac{K_{\mathrm{B}}{T}_{\mathrm{c}}}{q} $$

in which *K*_B_ is the Boltzmann constant, *T*_c_ is the cell temperature, and *q* is the elementary charge. For the tandem structure of dual-junction cell, the short-circuit current is determined by the smallest one of the subcell, and the open-circuit voltage is presented as the sum of subcells' voltages. To obtain high conversion efficiency, a current matching between the top GaInP cell and the bottom GaAs cell is needed. It has been reported that the light absorption and reflection of NWs is highly sensitive to the NW diameter due to the diameter dependent dispersion properties in the NWs [32,33]. To determine the ideal structure for current matching, a wide range of diameters (150 ~ 400 nm) are considered.

## Results and discussion

**Figure 3:**
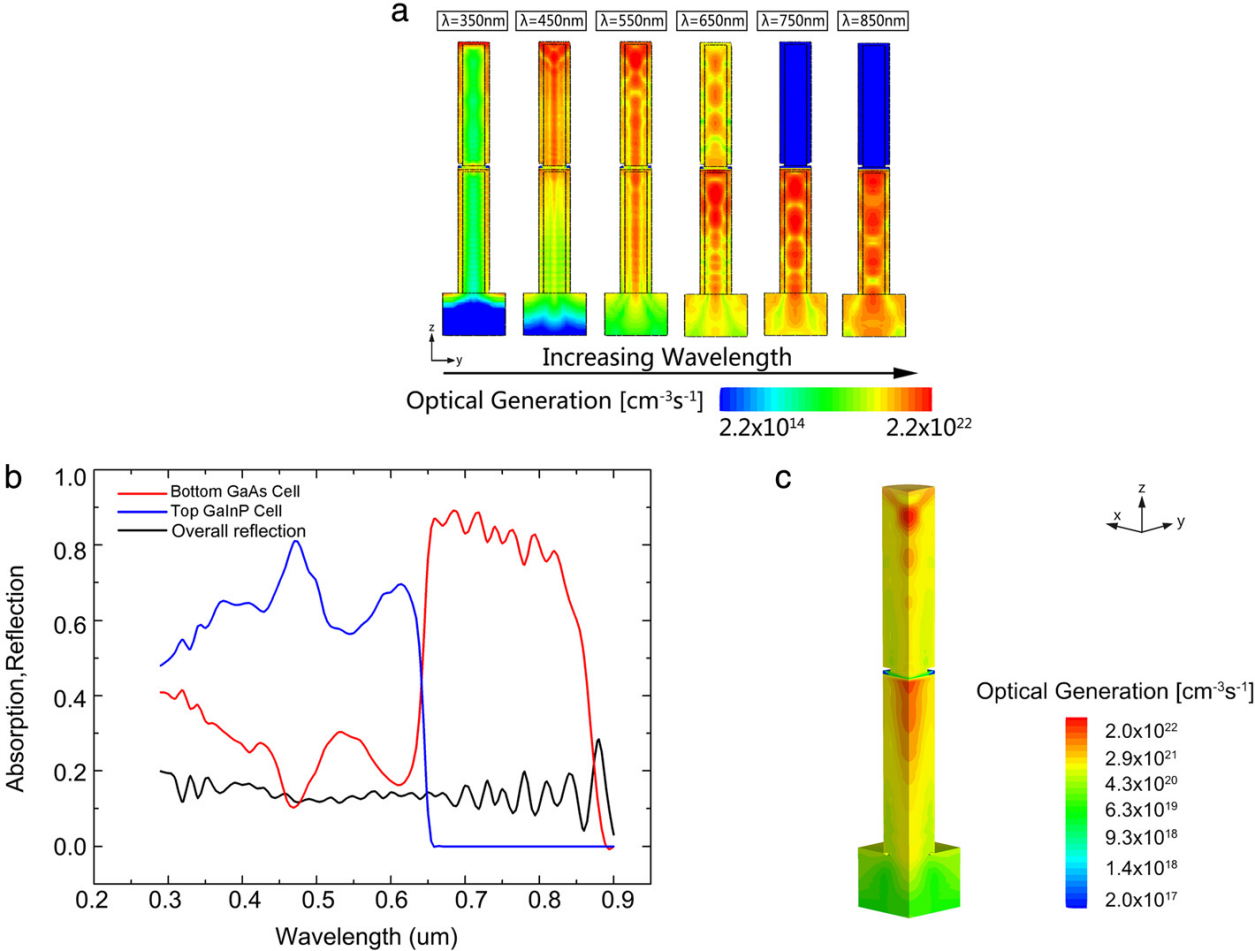
**Optical generation and total photogeneration profiles, absorbance of the subcells and overall reflectance. (a)** Optical generation profiles calculated by FDTD for wavelengths at 350, 450, 550, 650, 750, and 850 nm under 1 kW/m^2^. **(b)** The absorptance of the subcells and overall reflectance at a D/P ratio of 0.5. **(c)** The total photogeneration profiles in a quarter of the proposed structure.

Figure 3a displays vertical cross sections of optical generations through the center of NWs with increasing wavelengths under 1 kW/m^2^ illuminations. The result shows a clear separation of absorption spectrum. At *λ* = 350 nm, photocarriers are mainly generated at the surface of the NWs, showing a fairly short absorption length due to the high absorption capacity of GaInP and GaAs. At slightly longer wavelengths such as 450 nm, we can see that the majority of light is absorbed at the top GaInP cell. As wavelength increases, the optical generation becomes more spread through the NWs, while more and more light can be transmitted and absorbed by the bottom cell.

Figure 3b shows the absorption and reflection spectra of the simulated GaInP/GaAs solar cell at a fixed *D*/*P* ratio of 0.5. As can be seen in the spectra, different cells absorb light in different ranges, which shows a promising ability to achieve an excellent current matching when the subcells are axially connected. It can be seen that the total reflectance is lower than 0.2 from 290 to 900 nm due to the low effective refractive index, showing a good anti-reflection capacity. And the total absorptance for two junctions is higher than 80% which demonstrates the incident sunlight can be coupled into the nanowires efficiently with the excellent light trapping and anti-reflection.

Benefiting from the good light trapping and anti-reflection schemes, the incident sunlight can be efficiently absorbed by NWs at a low filling ratio. The total optical generation profiles in a quarter of the structure are shown in Figure 3c. Most of the photocarriers are generated in the first hundreds of nanometers of the NWs with a filling ratio of 0.196, implying an excellent optical absorption capacity in NWs.

**Figure 4:**
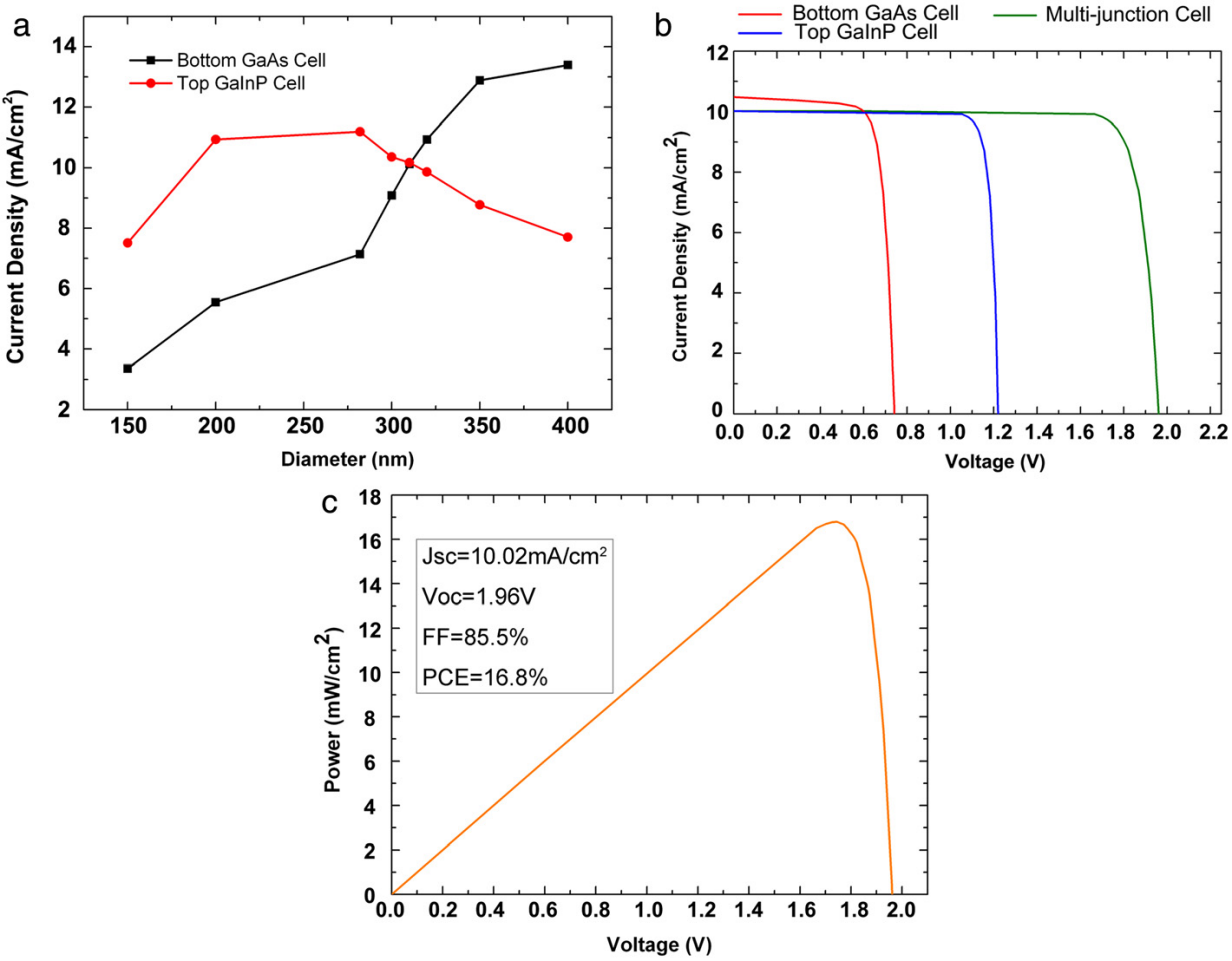
**Current density with different diameters and current-voltage and power-voltage of the proposed structure. (a)** The current density of the subcells as a function of diameters of shell NWs with a fixed *D*/*P* ratio of 0.5. **(b)** The current-voltage and **(c)** the power-voltage of the proposed axially connected NW core-shell p-n junctions with a NW diameter of 310 nm and *D*/*P* ratio of 0.5.

By tuning the diameters of NWs, the current matching is achieved at the NW diameter of 310 nm finally. The current densities for each subcell as a function of the NW diameter (150 to 400 nm) are shown in Figure 4a. For smaller diameters, the photocurrents are limited by the low light-trapping ability. Then, the photocurrents increase with the increasing diameter at first. When the diameter is further increased, the photocurrent in the top GaInP cell decreases due to the reduced absorption of dominant modes. More light not absorbed by the top cell is coupled into the bottom cell, resulting in a rapid increase of current density. And the simulated current-voltage characteristics of the subcells are shown in Figure 4b with an excellent current matching. The total *J*-*V* characteristic yields a short-circuit current (*J*_sc_) of 10.02 mA/cm^2^ and an open-circuit voltage (*V*_oc_) of 1.96 V. The power conversion efficiency (PCE) and the filling factor (FF) of the proposed structure, which can be obtained from the power-voltage characteristics shown in Figure 4c, are 16.8% and 0.855, respectively. In comparison, GaAs (GaInP) radial single junction devices with a length of 2 um under the same condition yields 13%, 21.3 mA/cm^2^, 0.811 V (12.8%, 12.3 mA/cm^2^, 1.26 V). In consideration that the material consumption is just 0.2 of the thin-film solar cells, the performance is comparable to the state-of-the-art GaInP/GaAs dual-junction solar cells (27%) under the illumination of 1 sun (AM 1.5G) [34]. A single GaAs-based solar cell has been recently reported to have a short-circuit current density over 160 mA/cm^2^ [35,36]. There is a main difference in the calculation of short-circuit current density (*J*_sc_) and efficiency between the single nanowire and nanowire arrays, that is, the area used to divide the short-circuit current (*I*_sc_) is the top surface area of nanowire in the single nanowire case, instead of the substrate area in the case of nanowire arrays. Therefore, the *J*_sc_ of nanowire array is always lower than the single nanowire device. The highest ever recorded efficiency of nanowire arrays is 13.8%, which is reported by Wallentin's work [18]. Fine adjustments of bandgap combination and more subcells with a lower bandgap such as Ge are believed to enhance the performance even further.

### Comparison with axial p-n junctions

**Figure 5:**
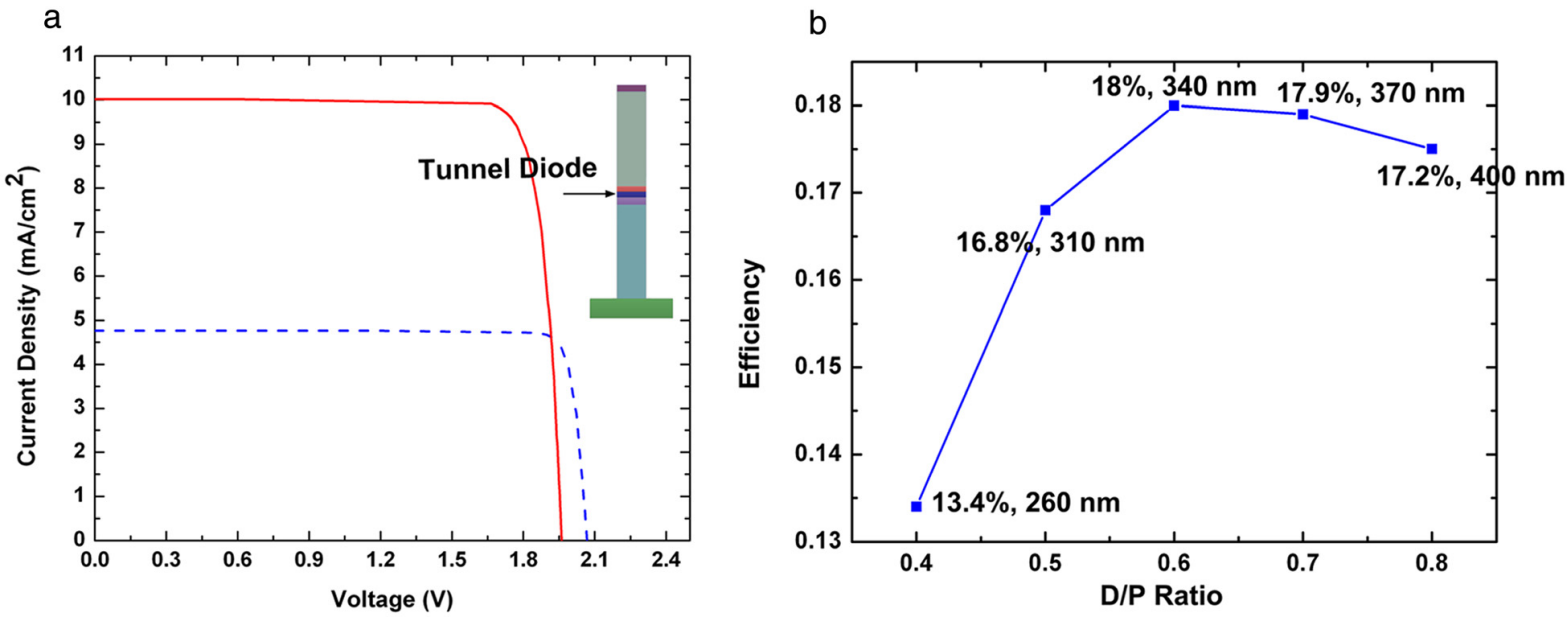
**Comparison of I-V characteristics and conversion efficiency with different D/P ratios. (a)** The comparison of I-V characteristics between the proposed structure and the axial p-n junctions. **(b)** The conversion efficiency as a function of the *D*/*P* ratio. The numbers between every point are the efficiency, and NW diameter when current matching is achieved.

To assess the advantage of core-shell p-n junction NW, the PV performance of the tandem axial p-n junctions on n-type GaAs substrate with the same volume and doping concentration is also simulated. For a relatively low loss of light when transmitted in the n-doped region, the NW axial p-n junction is employed with a short n-type region (100 nm) and a long p-type region (1,900 nm), at a fixed *D*/*P* ratio of 0.5. A coupled optoelectronic simulation and current matching which is same as the abovementioned are carried out to obtain the *J*-*V* characteristics. Figure 5a shows the comparison of *J*-*V* characteristics between the axial and core-shell p-n junctions. As can be seen from the figure, the axial one yields a higher *V*_oc_ (2.07 V) but a much lower *J*_sc_ (4.77 mA/cm^2^), which results in lower efficiency (8.87%). The higher *V*_oc_ may be explained by the larger depletion region in the axial p-n junctions. The width of the depletion region mainly depends on the material and doping concentration. However, the p-doped region (shell) in the radial p-n junctions is very thin, which may cause smaller depletion region. And the much lower *J*_sc_ can be explained by the large absorption area along the axial direction and efficient carrier collection in core-shell p-n junctions. For the majority of wavelengths absorbed in GaInP/GaAs, light is transmitted through the NW as shown in Figure 3a, which cannot be absorbed sufficiently by the axial p-n junction due to the small area and fixed position of depletion region. Subsequently, the short collection length in NW core-shell p-n junctions promote the efficient collection of the photocarriers, which provides a further higher current density.

### Device optimization

As mentioned above, the performance of the structure with a fixed *D*/*P* ratio of 0.5 is not optimal for the maximum efficiency. And the nanowire density has an important impact on the device performance [37]. In this section, the PV performance with different *D*/*P* ratio is simulated as shown in Figure 5b. With increasing *D*/*P* ratio, the conversion efficiency increases first and then decreases, obtaining a highest efficiency of 18% at a *D*/*P* ratio of 0.6 and NW diameter of 340 nm. The *J*_sc_ is 11.1 mA/cm^2^ and *V*_oc_ is 1.94 V. This phenomenon can be understood by the increased light reflection at the top surface of NWA. The effective refractive index of the NW arrays increases as *D*/*P* ratio increases, resulting in the decline of anti-reflection capacity. Furthermore, in consideration to the fact that GaAs and Ga_0.5_In_0.5_P is lattice matched, high-quality nanowires with little defect are expected to be achieved. Thus, a larger *τ*_SRH_ of 10 ns can be adopted and a further improved efficiency of 19.9% (*J*_sc_ = 11.5 mA/cm^2^ and *V*_oc_ = 2.04 V) can be obtained with a *D*/*P* ratio of 0.6 after the current matching. There is still some room for improvement in the optimization of the device, and a more precise and optimal estimate of efficiency will be presented in our future work.

## Conclusions

In summary, a novel NW structure for solar cells that axially connects core-shell p-n junctions with different bandgaps is proposed in this paper. A coupled three-dimensional optoelectronic simulation is used to evaluate the performance of the proposed structure. FDTD method is employed in the optical simulation. An excellent current matching of subcells is achieved by adjusting the diameter of the NWs. The simulation results reveal a high conversion efficiency of 16.8% at a low filling ratio of 0.196. Comparison with tandem axial p-n junctions is made, indicating that core-shell p-n junctions have a much higher photocurrent under the same conditions. Some device optimization is made and a maximum efficiency of 19.9% is achieved by tuning the *D*/*P* ratio of the NW arrays and *τ*_SRH_. By adopting optimized bandgaps or more junctions, the performance of the proposed structure is expected to be further improved.

## Abbreviations

EMW: electromagnetic wave
FDTD: finite-difference time-domain
FF: filling factor
J_sc_: short-circuit current
NWs: nanowires
PCE: power conversion efficiency
PML: perfect matching layer
PV: photovoltaic
TE: transverse electric
TM: transverse magnetic
VLS: vapor-liquid-solid
V_oc_: open-circuit voltage
